# Identifying prior signals of bipolar disorder using primary care electronic health records: a nested case–control study

**DOI:** 10.3399/BJGP.2022.0286

**Published:** 2024-02-06

**Authors:** Catharine Morgan, Darren M Ashcroft, Carolyn A Chew-Graham, Matthew Sperrin, Roger T Webb, Anya Francis, Jan Scott, Alison R Yung

**Affiliations:** Faculty of Biology, Medicine and Health, Manchester Academic Health Science Centre, National Institute for Health and Care Research (NIHR) Greater Manchester Patient Safety Research Collaboration, NIHR School for Primary Care Research, University of Manchester, Manchester, UK.; Centre for Pharmacoepidemiology and Drug Safety, Faculty of Biology, Medicine and Health, Manchester Academic Health Science Centre, NIHR Greater Manchester Patient Safety Research Collaboration, NIHR School for Primary Care Research, University of Manchester, Manchester, UK.; School of Medicine, Keele University, Keele, UK.; School of Health Sciences, Division of Informatics, Imaging & Data Sciences, Faculty of Biology, Medicine and Health, Manchester Academic Health Science Centre, NIHR Greater Manchester Patient Safety Research Collaboration, NIHR School for Primary Care Research, University of Manchester, Manchester, UK.; Centre for Mental Health & Risk, Faculty of Biology, Medicine and Health, Manchester Academic Health Science Centre, NIHR Greater Manchester Patient Safety Research Collaboration, NIHR Manchester Biomedical Research Centre, University of Manchester, Manchester, UK.; Centre for Psychology and Mental Health, School of Health Sciences, University of Manchester, Manchester, UK.; Institute of Neuroscience, Newcastle University, Newcastle-upon-Tyne, UK; Department of Mental Health, Norwegian University of Science and Technology, Trondheim, Norway; Department of Mental Health, Université de Paris, Paris, France; Brain and Mind Centre, University of Sydney, Sydney, Australia.; Institute for Mental and Physical Health and Research Translation, Deakin University, Geelong, Australia; emeritus professor of psychiatry, Centre for Psychology and Mental Health, School of Health Sciences, University of Manchester, Manchester, UK.

**Keywords:** bipolar disorder, case–control studies, electronic health records, primary health care, prodromal symptoms, signs and symptoms

## Abstract

**Background:**

Bipolar disorders are serious mental illnesses, yet evidence suggests that the diagnosis and treatment of bipolar disorder can be delayed by around 6 years.

**Aim:**

To identify signals of undiagnosed bipolar disorder using routinely collected electronic health records.

**Design and setting:**

A nested case–control study conducted using the UK Clinical Practice Research Datalink (CPRD) GOLD dataset, an anonymised electronic primary care patient database linked with hospital records. ‘Cases’ were adult patients with incident bipolar disorder diagnoses between 1 January 2010 and 31 July 2017.

**Method:**

The patients with bipolar disorder (the bipolar disorder group) were matched by age, sex, and registered general practice to 20 ‘controls’ without recorded bipolar disorder (the control group). Annual episode incidence rates were estimated and odds ratios from conditional logistic regression models were reported for recorded health events before the index (diagnosis) date.

**Results:**

There were 2366 patients with incident bipolar disorder diagnoses and 47 138 matched control patients (median age 40 years and 60.4% female: *n* = 1430/2366 with bipolar disorder and *n* = 28 471/47 138 without). Compared with the control group, the bipolar disorder group had a higher incidence of diagnosed depressive, psychotic, anxiety, and personality disorders and escalating self-harm up to 10 years before a bipolar disorder diagnosis. Sleep disturbance, substance misuse, and mood swings were more frequent among the bipolar disorder group than the control group. The bipolar disorder group had more frequent face-to-face consultations, and were more likely to miss multiple scheduled appointments and to be prescribed ≥3 different psychotropic medication classes in a given year.

**Conclusion:**

Psychiatric diagnoses, psychotropic prescriptions, and health service use patterns might be signals of unreported bipolar disorder. Recognising these signals could prompt further investigation for undiagnosed significant psychopathology, leading to timely referral, assessment, and initiation of appropriate treatments.

## Introduction

Bipolar disorders are serious mental illnesses characterised by instability in mood and rest–activity rhythms. The lifetime prevalence of bipolar disorders (which comprise bipolar disorder type I and type II, and spectrum disorders) is between 1.0% and 3.7%,^1^^,^^2^ and the peak age range for onset is 18–22 years.^2^ Despite the clinical, social, and economic burden of bipolar disorder, there is a delay between the early manifestations of bipolar disorder and its diagnosis and/or treatment of about 6 years.^3^^,^^4^ In a study of individuals with unipolar depression attending primary care services, at least one in 30 were identified as having undiagnosed bipolar disorder.^4^ In a recent UK study, 10% of 233 individuals prescribed antidepressants in primary care were found to have previously undiagnosed bipolar disorder.^5^ Similar results of underdiagnosis of bipolar disorder in primary care were found in a meta-analysis using structured clinical interview and screening tools for identification.^6^

Delayed diagnosis of bipolar disorder is associated with poor social adjustment, multiple hospital admissions,^7^ elevated risk of self-harm, suicide, and interpersonal violence,^8^ and greatly raised prevalence of cardiovascular, endocrine/metabolic, and neurological conditions.^9^ Missed or delayed diagnosis of bipolar disorder might also result in inappropriate prescribing, such as the use of antidepressant monotherapy, which in turn might increase the risk of drug-induced hypomania or drug-induced mania,^10^ or trigger rapid cycling.^11^

Despite the poor outcomes related to delayed diagnosis, screening tools such as the Hypomania Checklist^12^ and the Mood Disorder Questionnaire^13^ are found to have varied performance within different healthcare settings, with high false-positive rates.^14^^,^^15^ Furthermore, one of the best predictors of developing bipolar disorder, a family history of bipolar disorder (especially in first-degree relatives), is not routinely entered in patients’ primary care records.

**Table:** How this fits in

Delayed diagnosis and treatment of bipolar disorder of between 6 and 10 years leads to adverse patient outcomes. To the authors’ knowledge, no published studies examine the timings of early signals of bipolar disorder in a primary care setting and/or use electronic health records. The current study found that routinely collected data identified the following early signals of undiagnosed bipolar disorder: previous depressive episodes, sleep disturbance, substance misuse, those receiving ≥3 different psychotropic medication classes in a year, escalating self-harm, twice as many face-to face consultations, and missing scheduled appointments. Awareness of collective early signals can be used to prompt consideration of bipolar disorder and offer timelier referral for specialist assessment of a bipolar disorder diagnosis and initiation of appropriate treatment.

Retrospective studies indicate that individuals diagnosed with bipolar disorder might experience a range of antecedent psychopathology, including childhood anxiety and adolescent depression. In addition, personality disorders, psychotic symptoms, and/or a subthreshold psychotic episode might occur before a bipolar disorder diagnosis.^7^^,^^16^^–^^19^ In summary, to the authors’ knowledge, there are currently no screening instruments or risk calculators that can assist GPs in making a timely bipolar disorder diagnosis. However, this does not mean that it is impossible to identify individuals who have characteristics that could indicate the presence of unrecognised bipolar disorder and who might benefit from referral for a diagnostic assessment. As such, the purpose of this descriptive study was to identify potential signals of bipolar disorder from primary care electronic health records among individuals who were subsequently diagnosed with bipolar disorder, and to compare them with individuals who did not receive a bipolar disorder diagnosis during this time. In addition, utilisation of a large cohort of primary care electronic health records with a long historic observation period enabled descriptive exploration of signals over time to facilitate operationalising them before a bipolar disorder diagnosis.

## Method

### Data sources

The Clinical Practice Research Datalink (CPRD) GOLD dataset holds information from general practices in all regions of the UK. It covers approximately 7% of all individuals registered with a general practice and is broadly representative of the national population in terms of age, sex, and ethnicity.^20^ The primary care electronic health records hold routinely collected information and patient–GP interactions pertaining to symptoms, diagnoses, prescribed medication, and referrals to secondary care services.

**Figure 1.**
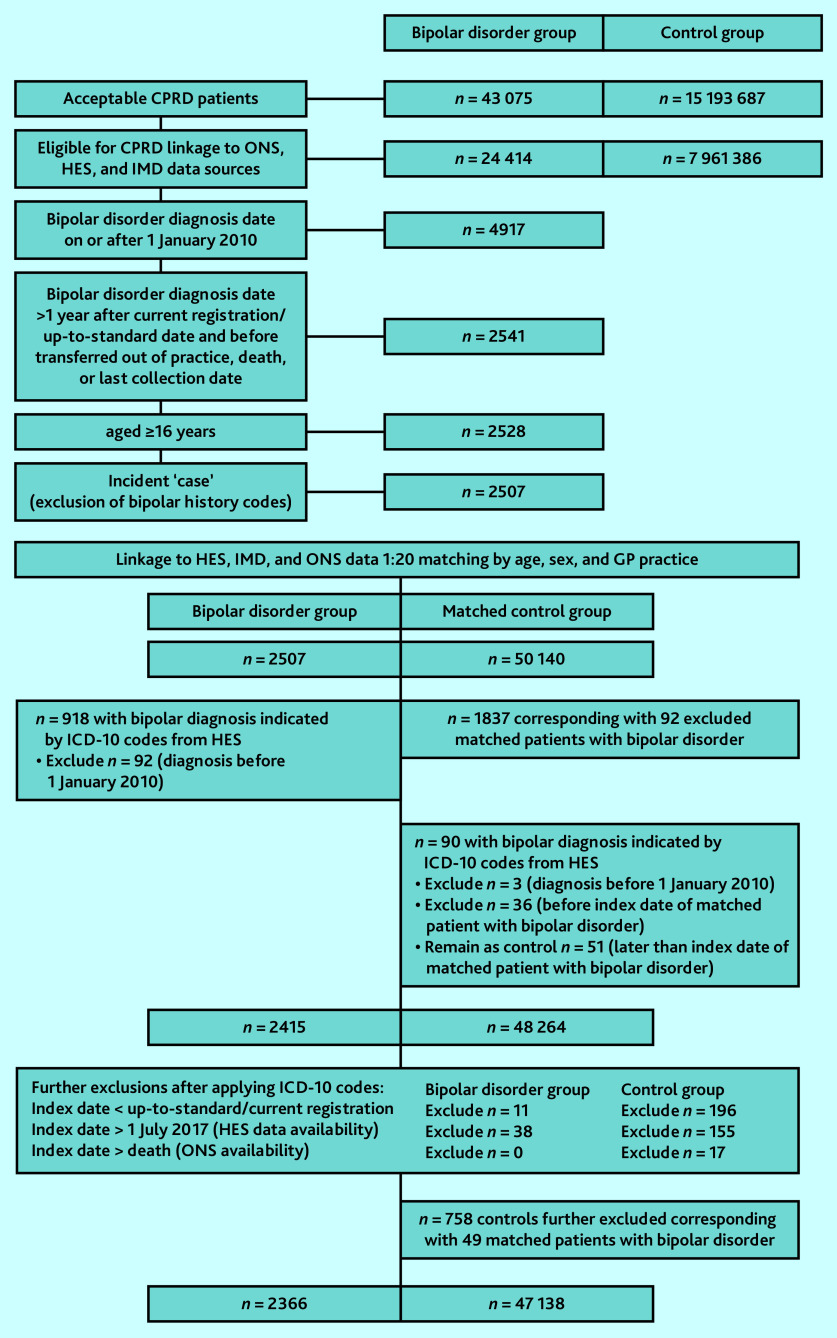
Flowchart summarising the derivation and delineation of the study cohort from interlinked CPRD GOLD and HES datasets. CPRD = Clinical Practice Research Datalink. HES = Hospital Episode Statistics. IMD = Index of Multiple Deprivation. ONS = Office for National Statistics.

For a subset of English practices participating in the CPRD linkage scheme, which included 56.7% (bipolar disorder group) and 52.4% (control group) of eligible patients for linkage, secondary care clinical data were obtained through linkage to Hospital Episode Statistics (HES), including International Classification of Diseases, 10th revision (ICD-10) diagnostic coding of inpatient episodes and emergency department attendance. Interlinkage of primary and secondary data sources enhanced ascertainment of individuals with incident bipolar disorder (Figure 1) and events related to antecedent signals of bipolar disorder. The area-level Index of Multiple Deprivation (2015) was obtained as a deprivation measure derived from a combination of socioeconomic indicators based on practice postcode.

Following a review of the literature and input from clinical experts and members of the authors’ lived experience advisory panel, a list of candidate symptoms and signals that might precede bipolar disorder onset was considered and specified according to primary care Read codes (CPRD) and secondary care ICD-10 codes (HES — including emergency department attendance). Complete information on medication prescribed in general practice was extracted from patient records held in the CPRD. Health events of interest included indications of:

- mental health or recorded diagnosis of: depression (and related symptoms); anxiety disorders; psychotic disorders including schizophrenia, personality disorders, suicidal ideation, and self-harm; drug and alcohol misuse; anger and aggression; mood swings; and sleep disturbance;
- prescribed medication: antidepressants; antipsychotics; benzodiazepines; Z-drugs; gabapentoids; mood stabilisers; strong opioids; and the number of psychotropic medication classes prescribed during each year of observation; and
- health service interactions: consultations with a GP or practice nurse; non-attendance at scheduled appointments; referral to mental health services; emergency department attendances; and subsequent admissions.

### Study design

A case–control study design was implemented. The first bipolar disorder event recorded in the electronic health record was identified either through a relevant primary care Read code (CPRD data) or ICD-10 codes (F30–F31 inclusive) from hospital admission records, whichever code was dated earliest. All adults aged ≥16 years with an incident diagnosis between 1 January 2010 and 31 July 2017 were included.

The definition of bipolar disorder included manic episodes, and the classification was subject to clinical review by a senior researcher with expertise in general practice (the third author) and independently by a senior academic psychiatrist (the senior author). The date pertaining to the incident diagnostic code was set as the index date and the ‘cases’ (individuals in the bipolar disorder group) were then matched by age, sex, and registered general practice with up to 20 ‘controls’ without a recorded diagnosis of bipolar disorder on the index date of the corresponding matched ‘case’. Matching on registered general practice ensured balance between ‘case’ and ‘control’ patients on numerous unmeasured practice-level and other system-level factors. This effectively removed such influences from the investigation, thereby enabling the authors to examine patient-specific factors independent of these extraneous factors. Figure 1 shows the study flowchart, including cohort recruitment and derivation of first bipolar disorder diagnosis date from both CPRD and HES data sources.

### Analysis

For each health event examined, the annual episode incidence rate per 1000 person–years (and its 95% confidence interval [CI]) was estimated over time for each year before the index date for individuals with bipolar disorder and their matched controls. The signals were presented as binary indicator variables and counted as being ‘present’ or ‘absent’ once only in each year period before the index date from the date the individual registered with their general practice for at least a year (Supplementary Table S1).

Odds ratios (ORs, and their 95% CIs) were calculated for symptoms and signals, measuring the association between each health event and having a recorded bipolar disorder diagnosis. The OR indicates, for patients with a recorded bipolar disorder code (the bipolar disorder group), how many more times their odds were of having the prior symptom or signal of interest versus those without a bipolar disorder diagnosis (the control group). ORs were presented in year ranges of <1 year, 1 to <3 years, 3 to <5 years, and at 5 to <10 years before the index date. Each dichotomous event variable was entered into their own model for each year range using conditional logistic regression, thereby accounting for the matched design.

The number of classes of psychotropic medication within a given year, non-attendance at scheduled appointments, referrals to mental health services, and emergency department attendance were aggregated annually before the index date and analysed as count data within each year. Face-to-face patient–GP consultations were presented as median counts and interquartile ranges (IQRs).

All code lists applied in this study are published online (https://clinicalcodes.rss.mhs.man.ac.uk/medcodes/article/201). All analyses were conducted using Stata/SE (version 14.2). Details of Stata analysis code can be found in Supplementary Information S1.

## Results

A total of 2366 people diagnosed with bipolar disorder and 47 138 matched control patients were included in the analysis (60.4% female: *n* = 1430/2366 with bipolar disorder and *n* = 28 471/47 138 without). The median age at the date of index diagnosis was 40 years (IQR 30–53).

### Mental health, self-harm, and addiction

**Figure 2.**
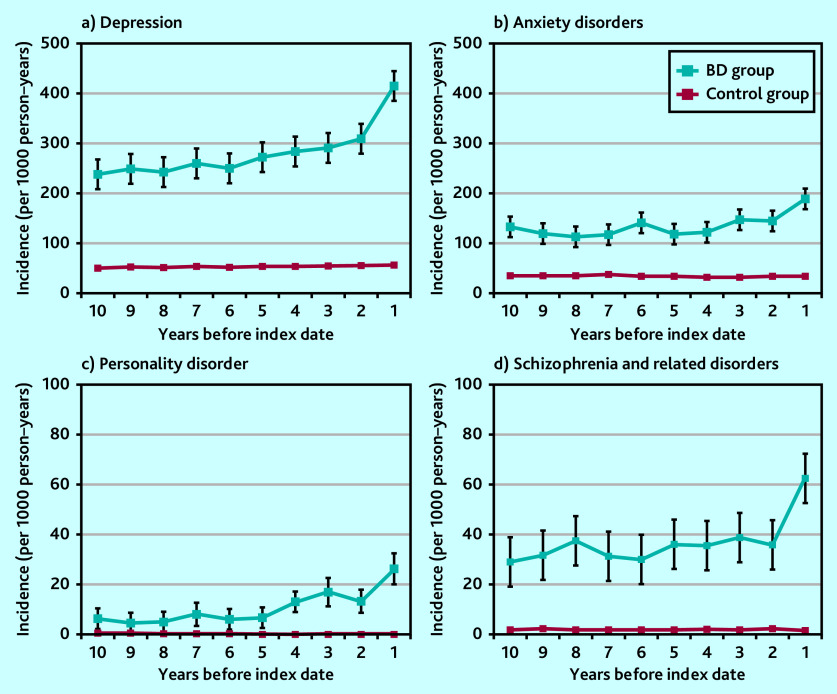
Annual episode incidence of a) depression; b) anxiety disorders; c) personality disorders; and d) schizophrenia and related disorders in patients with bipolar disorder (bipolar disorder group) and without bipolar disorder (control group) from 10 years before the bipolar disorder diagnosis was made. BD = bipolar disorder.

As early as 10 years before bipolar disorder diagnosis there was an incidence of 240 per 1000 person–years of recorded diagnosis of depression and depressive symptoms among the bipolar disorder group. As shown in Figure 2a, depressive episodes and symptoms increased from 6 years before the index date. Those diagnosed with bipolar disorder were more likely to have had a record of anxiety disorders, with an increased incidence 5 years before the index date, albeit to a less degree than for recorded diagnosis of depression (Figure 2b). The odds of someone with a bipolar disorder diagnosis also having a prior record of a personality disorder diagnosis was 28 times larger than the odds for someone without a bipolar disorder diagnosis 3 to <5 years before the index date; the odds were 26 times larger for a prior diagnosis of schizophrenia and related disorders (Table 1 and Figures 2c and 2d).

**Table 1.** ORs for people diagnosed with bipolar disorder (versus control group) having an additional psychiatric illness diagnosis, symptom, or behaviour signal, or prescribed psychotropic medication recorded before index date

**Characteristic**	**Number of years before bipolar disorder diagnosis date, odds ratio (95% CI)**			

	**<1 year**	**1 to <3 years**	**3 to <5 years**	**5 to <10 years**
**Illnesses, symptoms, and behaviours**				
Depression and/or depressive symptoms	13.2 (12.0 to 14.6)	8.5 (7.8 to 9.3)	6.9 (6.2 to 7.7)	6.3 (5.6 to 7.1)
Personality disorders	51.8 (32.4 to 82.9)	31.9 (21.0 to 48.3)	28.1 (14.1 to 55.9)	13.4 (7.7 to 23.2)
Schizophrenia and related disorders	77.6 (54.1 to 111.4)	25.2 (19.5 to 32.6)	26.1 (18.4 to 36.9)	24.1 (17.2 to 33.7)
Anxiety disorders	7.4 (6.6 to 8.4)	5.3 (4. 8 to 6.0)	4.0 (3.5 to 4.5)	4.1 (3.6 to 4.7)
Mood swings	45.2 (33.4 to 61.2)	14.4 (10.6 to 19.6)	6.3 (4.0 to 9.7)	6.1 (4.2 to 8.7)
Sleep disturbance	5.5 (4.5 to 6.5)	4.5 (3.9 to 5.2)	3.5 (2.9 to 4.2)	3.2 (2.7 to 3.8)
Self-harm and suicidal ideation	28.2 (22.5 to 35.2)	11.7 (9.6 to 14.3)	8.4 (6.5 to 11.0)	8.8 (7.0 to 11.1)
Drug misuse	7.8 (5.7 to 10.8)	4.6 (3.4 to 6.3)	6.4 (4.5 to 9.0)	4.3 (3.0 to 6.0)
Alcohol misuse	5.8 (4.4 to 7.5)	4.1 (3.3 to 5.2)	4.5 (3.3 to 6.2)	4.5 (3.3 to 6.1)
Anger or aggression	15.7 (11.4 to 21.7)	7.7 (5.8 to 10.3)	6.6 (4.6 to 9.5)	4.5 (3.3 to 6.1)

**Prescribed medication**				
Antidepressants	14.4 (13.1 to 15.8)	10.2 (9.3 to 11.2)	8.3 (7.5 to 9.2)	6.0 (5.4 to 6.8)
Antipsychotics	23.1 (20.7 to 25.8)	11.0 (9.9 to 12.3)	7.5 (6.6 to 8.6)	5.2 (4.5 to 5.9)
Benzodiazepines	9.1 (8.1 to 10.1)	5.2 (4.7 to 5.8)	4.0 (3.5 to 4.6)	3.4 (2.9 to 3.8)
Z-drugs	11.6 (10.2 to 13.1)	6.6 (5.8 to 7.5)	6.4 (5.5 to 7.4)	4.6 (3.9 to 5.4)
Mood stabilisers (excluding lithium)^a^	16.9 (14.6 to 19.7)	9.0 (7.6 to 10.7)	6.3 (5.1 to 7.8)	4.4 (3.4 to 5.7)
Gabapentinoids	3.6 (3.0 to 4.5)	2.9 (2.4 to 3.7)	2.5 (1.8 to 3.4)	2.1 (1.4 to 3.1)
Strong opioids	2.6 (2.2 to 3.1)	2.3 (2.0 to 2.7)	2.0 (1.7 to 2.4)	1.7 (1.4 to 2.1)

a *Assumption of lithium prescription as an indication of bipolar disorder diagnosis and therefore excluded. OR = odds ratio.*

**Figure 3.**
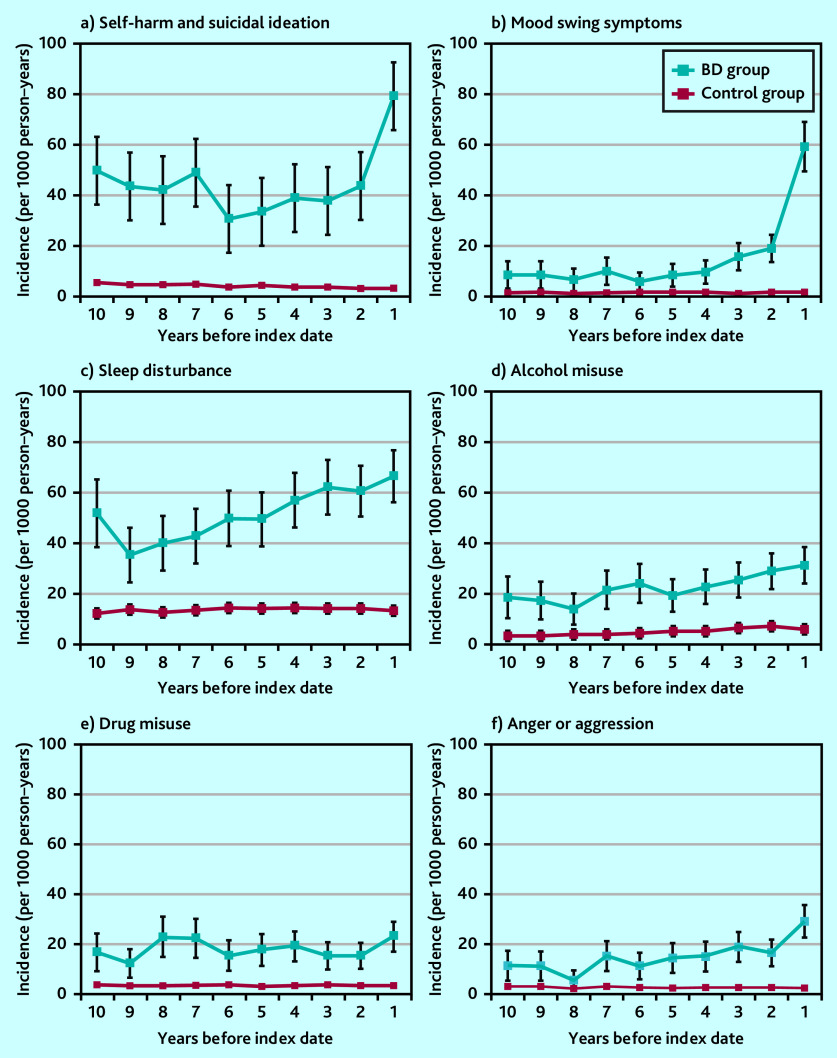
Annual episode incidence of a) self-harm and suicidal ideation; b) mood swings; c) sleep disturbance; d) alcohol misuse; e) drug misuse; and f) anger or aggression in patients with bipolar disorder (bipolar disorder group) and without bipolar disorder (control group) from 10 years before the bipolar disorder diagnosis was made. BD = bipolar disorder.

Self-harm and suicidal ideation episodes increased in frequency in the period leading up to a bipolar disorder diagnosis. They were noted as early as 10 years before the index diagnosis. Those diagnosed with bipolar disorder were eight times more likely to have harmed themselves or had suicidal thoughts recorded in their notes than those without a bipolar disorder diagnosis 3 to <5 years before the index date (Table 1 and Figure 3a). Mood swings also occurred more frequently before a bipolar disorder diagnosis, with a marked increase 2 years before the index date (Figure 3b). Sleep disturbance showed a pattern of rising incidence over a long time period (of at least 10 years) before the index diagnosis (Figure 3c).

Drug and alcohol misuse were also more frequently recorded for individuals subsequently diagnosed with bipolar disorder compared with the control group (Figures 3d and 3e). Higher drug and alcohol misuse rates were present 10 years before diagnosis. In the 1 year before the index diagnosis date, the bipolar disorder group were over eight and six times more likely to experience drug or alcohol misuse, respectively, than those in the control group (Table 1 and Figures 3d and 3e). The pattern of coding for anger and aggression was similar (Table 1 and Figure 3f). In the 1 year before diagnosis, those with a bipolar disorder diagnosis were over 15 times more likely to have anger or aggression issues recorded than those without a bipolar disorder diagnosis.

### Psychotropic medication prescribing

**Figure 4.**
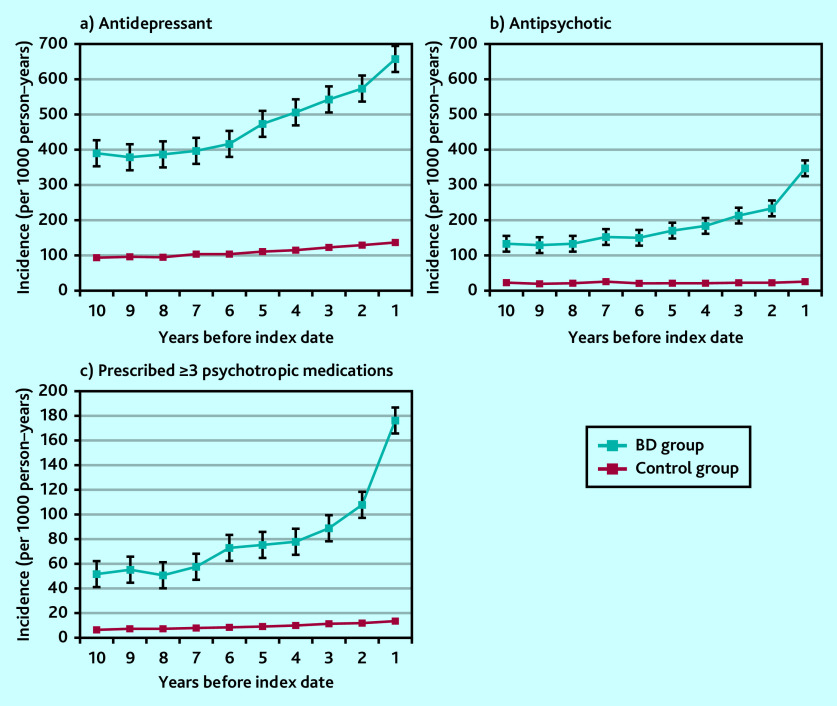
Annual episode incidence of prescribing a) an antidepressant; b) antipsychotic medication; and c) ≥3 psychotropic medications (includes antidepressants, antipsychotics, benzodiazepines, Z-drugs, mood stabilisers, gabapentin, pregabalin, and strong opioids) in patients with bipolar disorder (bipolar disorder group) and without bipolar disorder (control group) from 10 years before the bipolar disorder diagnosis was made. One prescription in each medication category counted only once per annum. BD = bipolar disorder.

Antidepressants and antipsychotics were much more widely prescribed than any other psychotropic medication type (Figures 4a and 4b). In particular, antidepressants were more frequently prescribed in individuals who were later diagnosed with bipolar disorder compared with the control group. High frequency was observed as early as 10 years before diagnosis, markedly increasing ≤6 years before index bipolar disorder diagnosis date. At 3 to <5 years before the index date, those receiving a bipolar disorder diagnosis were eight times more likely to be prescribed an antidepressant (Table 1), and four and six times more likely to be prescribed a benzodiazepine or a Z-drug, respectively, than those without a bipolar disorder diagnosis recorded (Supplementary Figure S1).

Differences were also seen in the number of psychotropic medication classes prescribed. Comparing those with a bipolar disorder diagnosis and those without, those in the bipolar disorder group were eight times more likely to be prescribed ≥3 different classes of psychotropic medication during the same year compared with those prescribed ≤2 different classes of psychotropic medication (OR 8.4, 95% CI = 6.8 to 10.6; *P*<0.001) (Figure 4c). This high likelihood was seen in the 2 years before diagnosis but also evident across the preceding 6-year period.

### Health service use

Statistically significant differences were observed between the bipolar disorder and control groups in the median number of consultations per year before the index date, although large variability around the median was observed (Supplementary Figure S2). At 3 to <5 years before the diagnosis date the median number of consultations was 8 (IQR 2–17) for the bipolar disorder group versus 4 (IQR 1–10) for the control group (Mann–Whitney test, *P*<0.001) (Table 2). At this time, those with a recorded bipolar disorder diagnosis were four times more likely to have missed ≥6 scheduled appointments compared with the control group (Table 2).

**Table 2.** Service interaction before index date among patients with bipolar disorder (bipolar disorder group) and without bipolar disorder (control group)

**Characteristic**	**Number of years before bipolar disorder diagnosis date**			

	**<1 year**	**1 to <3 years**	**3 to <5 years**	**5 to <10 years**
**Number of face-to-face consultations, median (IQR)**				
Bipolar disorder group	21 (12–32)	12 (5–22)	8 (2–17)	2 (0–8)
Matched control group	6 (2–13)	5 (2–12)	4 (1–10)	1 (0–5)

**Number of missed appointments, OR (95% CI)**				
1	2.09 (1.89 to 2.31)	1.89 (1.69 to 2.11)	1.70 (1.50 to 1.94)	1.42 (1.16 to 1.72)
2–3	4.34 (3.86 to 4.89)	2.83 (2.43 to 3.29)	2.50 (2.07 to 3.02)	2.04 (1.49 to 2.80)
4–5	7.29 (5.87 to 9.04)	3.91 (2.90 to 5.27)	5.36 (3.70 to 7.75)	4.39 (2.13 to 9.03)
≥6	12.63 (9.28 to 17.19)	7.38 (4.79 to 11.35)	4.42 (2.41 to 8.11)	3.64 (0.81 to 16.40)

**Number of ED attendances,^a^ OR (95% CI)**				
1	2.11 (1.82 to 2.46)	1.60 (1.36 to 1.89)	1.26 (1.05 to 1.51)	—
2–3	3.89 (3.23 to 4.68)	2.40 (1.92 to 3.00)	2.77 (2.22 to 3.48)	—
≥4	5.98 (4.30 to 8.33)	2.99 (1.81 to 4.93)	1.71 (0.86 to 3.38)	—

a *ED HES data source from April 2007, therefore there were too few individuals with >10 years of data to calculate OR for ED attendance at 10 years. ED = emergency department. HES = Hospital Episode Statistics. IQR = interquartile range. OR = odds ratio.*

## Discussion

### Summary

This study examined information that was routinely recorded in patients’ primary care electronic health records to identify the frequency and timing of potentially important and detectable signals of undiagnosed bipolar disorder. Depressive episodes and lifetime diagnoses of personality disorder and schizophrenia were frequently reported in the years before bipolar disorder diagnosis. Furthermore, several clinical characteristics showed an escalating pattern in the period proximal to bipolar disorder recognition.

Self-harm and suicidal ideation, although elevated at least 10 years before diagnosis, were markedly higher in the 2 years immediately preceding a bipolar disorder diagnosis. Sleep disturbance was present for many years before diagnosis, whereas mood swings appeared to be a later phenomenon, recorded more frequently 1–2 years before diagnosis. Drug or alcohol misuse and anger or aggression also occurred more commonly (than in the control group) among individuals who were later diagnosed with bipolar disorder.

Another potential indicator of undiagnosed bipolar disorder was psychotropic medication polypharmacy. Antidepressants, antipsychotics, and ≥3 psychotropic prescriptions within the same year were all more likely to be recorded in patients with undiagnosed bipolar disorder compared with the control group. Multiple GP consultations in 1 year and increasing frequency of not attending scheduled appointments for any reason also signalled undiagnosed bipolar disorder.

### Strengths and limitations

Identification of signals from information that is routinely entered in electronic health records is a major strength of this study. These datasets enabled investigation of a variety of potential signals including patterns of medication prescribing and patients’ interactions with health services, as well as prior symptoms and diagnoses. Furthermore, it was possible to implement a nested case–control design, sampled from a very large cohort of patients with bipolar disorder and control patients.

The study does, however, have some limitations. Researchers using electronic healthcare records are reliant on the Read codes applied in the course of clinical care or by practice administrators. These might not capture the rich contextual information useful for researchers. In addition, misclassification might occur because of code inputting and a degree of inconsistency in GP coding choices. Furthermore, the timing of bipolar disorder diagnosis is difficult to establish using only information that is contained in primary care records. However, by identifying relevant diagnostic codes from both primary care and linked secondary care records, it was possible to more accurately ascertain and date incident bipolar disorder diagnoses. Likely incomplete capture of family history of mental disorders, as one of the strongest risk factors for early onset of bipolar disorder,^21^ was an important limitation. GPs will more often report these details using free text, which, as a potential patient identifier, is not held by CPRD and therefore cannot be accessed for CPRD studies. Highlighting this limitation provides the opportunity to improve GP coding of family history within the electronic health record. Finally, use of a case–control design, although appropriate for this study’s purpose and research question, increased the likelihood of spurious association and selection bias. Therefore, the signals that are reported should be validated in a cohort study to enable more conclusive clinical interpretation.

### Comparison with existing literature

As previously reported, individuals who develop bipolar disorder experience a range of lifetime psychiatric comorbidities, especially antecedent anxiety disorders and depression.^22^^,^^23^ The raised incidence of depressive symptoms before bipolar disorder diagnosis was consistent with previous literature indicating that almost a quarter of patients with a previous diagnosis of unipolar major depressive disorder progress to bipolar disorder.^24^ However, it is difficult to determine whether the antecedent depression is part of the natural evolution of bipolar disorder (that is, where depression is the first manifestation and hypomania or mania occurs some years later)^24^^,^^25^ or whether evidence of hypomanic or manic symptoms or episodes have been missed,^7^ or indeed the individual has only sought help during long periods of depression. Thus, it is important to increase GP enquiries about periods of elation or increased activity levels in anyone seeking help for depression.

Antipsychotic medication prescription and diagnostic coding of schizophrenia and related disorders noted in patient records before bipolar disorder diagnosis is suggestive of the individual experiencing psychotic symptoms. Psychotic experiences often precede a diagnosis of bipolar disorder. Indeed, their presence increases the likelihood that individuals receive specialist assessments.^25^

A misdiagnosis as a condition other than bipolar disorder or missed diagnosis of bipolar disorder will lead to inappropriate clinical management. Overuse of antidepressants, especially without co-prescription of a mood stabiliser, might exacerbate affective symptoms thereby increasing the risk of drug-induced mania, switching between mania and hypomanic states, and emergence of rapid cycling, whereby the disorder accelerates with more mood shifts in a given timeframe.^26^ Although sleep disturbances are not unique to bipolar disorder, this was evident for many years before bipolar disorder diagnosis in the current study. Previous studies have shown sleep and circadian rhythm disturbances are common in patients with bipolar disorder,^27^ including during euthymia (that is, when the individual does not have an acute mood episode).^28^ Drug or alcohol misuse were also evident and escalated before bipolar disorder diagnosis. It is reported between 40% and 70% of individuals diagnosed with bipolar disorder experience substance misuse.^29^^,^^30^ Self-harm and suicidal ideation episodes, although elevated at least 10 years before diagnosis, were much more common in the 2 years before diagnosis. Previous studies have reported that delayed diagnosis or misdiagnosis are major contributors to elevated risk of dying by suicide among patients with bipolar disorder.^7^^,^^31^

### Implications for research and practice

For a person diagnosed with bipolar disorder, receiving the diagnosis earlier could reduce or prevent many harmful outcomes including antidepressant-induced rapid cycling,^11^ non-fatal self-harm and suicide,^31^^,^^32^ substance misuse,^29^^,^^30^ and interpersonal violence.^8^

Raising awareness among GPs, many of whom might lack understanding about the course of bipolar disorder and the associated signals,^33^ will improve recognition. Therefore, using the electronic health record as a prompt to process the signals cumulatively and highlight a probable bipolar disorder diagnosis to trigger further assessment when continuity of care is not always possible would be a helpful resource. It might also provide a useful addition to present screening tools to further enhance diagnostic specificity to bipolar disorder. In addition, the GP would have evidence from a patient’s electronic health record to support a referral to specialist care where the diagnosis of bipolar disorder is made.

Secondary mental health services, however, need to be responsive and willing to rapidly assess patients referred by GPs. National Institute for Health and Care Excellence guidance CG185 outlines the recognition of bipolar disorder in primary care for consideration of referral when patients present with depression along with previous periods of *‘overactivity or disinhibited behaviour’* lasting ≥4 days.^34^ In practice, however, this often does not occur because of high thresholds for acceptance to a community mental health team.^35^ Optimising the service pathway to improve the interface between primary and secondary care is essential. The roll-out of the Mental Health Implementation Plan^36^ in April 2021 offers a more integrated approach of primary and secondary care. It is also an opportunity to reduce barriers with more flexible care and to reassess the referral criteria threshold for possible bipolar disorder.

In conclusion, this study has identified potentially important signals within the primary care record that might prompt a GP to consider a diagnosis of bipolar disorder and support referral to secondary care. Additional work is needed to establish the specificity of these signals collectively, the sequential and concurrent nature of the signals, and their practical and acceptable use to both patients and GPs in primary care. This proactive approach could ensure that individuals receive timelier referral, intervention, and effective treatment to prevent a harmful impact on their mental and physical health.

## Data

The data are study specific and given by permission and application to CPRD, subject to license. Study data are therefore unavailable to be shared.
